# 肺部肿瘤热消融联合免疫治疗现状与进展

**DOI:** 10.3779/j.issn.1009-3419.2022.102.09

**Published:** 2022-04-20

**Authors:** 宝东 刘

**Affiliations:** 100053 北京，首都医科大学宣武医院胸外科 Department of Thoracic Surgery, Xuanwu Hospital, Capital Medical University, Beijing 100053, China

**Keywords:** 肺肿瘤, 热消融, 免疫治疗, Lung neoplasms, Thermal ablation, Immunotherapy

## Abstract

近来的研究表明，肿瘤免疫微环境与肿瘤的进展、转移、复发以及对治疗的反应密切相关；一些免疫治疗也给肿瘤患者带来了希望。然而，肿瘤免疫治疗存在疗效的不确定性和一定的副作用，为了增强其疗效，肿瘤免疫治疗联合肿瘤热消融的研究开始出现。肿瘤热消融具有微创、恢复快、安全、并发症少、适形、效果可靠、可重复、费用低等优点，现已成为继手术、放疗、药物治疗之后的肿瘤第四大治疗手段。它可以直接杀死肿瘤细胞并通过多种机制调节免疫系统，虽然相应的机制尚不清楚，但是联合肿瘤免疫治疗已被建议用于治疗几种实体恶性肿瘤。本文对肿瘤免疫治疗、肿瘤热消融、肺部肿瘤热消融联合肿瘤免疫的现状和进展进行了简要综述，并期待对肿瘤热消融联合肿瘤免疫的有效性和安全性的进一步研究。

## Abstract (en)

Recent studies have shown that tumor immune microenvironment is closely related to tumor progression, metastasis, recurrence and response to treatment. Some immunotherapies also offer hope for cancer patients. However, the efficacy of tumor immunotherapy is uncertain and has some side effects. In order to enhance its efficacy, tumor immunotherapy combined with tumor thermal ablation has been studied. Thermal ablation has the advantages of minimally invasive, rapid recovery, safety, fewer complications, conformation, reliable effect, repeatable, low cost, and has become the fourth tumor treatment measure after surgery, radiotherapy, and drug therapy. It can directly kill tumor cells and modulate the immune system through a variety of mechanisms, although the corresponding mechanisms are not well understood, but combined tumor immunotherapy has been proposed to treat several solid malignancies. In this review, the current status and progress of thermal ablation combined with immunotherapy for lung tumor were reviewed, and further studies on the efficacy and safety of thermal ablation combined with immunotherapy were expected.

肿瘤免疫（immuno-oncology, IO）联合肿瘤介入（interventional oncology, IO）（IO+IO）是目前临床研究的热点^[1-6]^。肿瘤免疫是研究肿瘤的抗原、机体的免疫功能与肿瘤发生、发展的相互关系，机体对肿瘤的免疫应答及其抗肿瘤免疫效应的机制、肿瘤的免疫诊断和免疫治疗的科学^[7]^。本文讨论的肿瘤介入主要是肿瘤热消融，包括射频消融（radiofrequency ablation, RFA）、微波消融（microwave ablation, MWA）和冷冻消融（cryoablation）三项技术。

## 1 肿瘤免疫

在肿瘤的免疫机制中，涉及到肿瘤抗原的免疫原性、机体的免疫监视和肿瘤的免疫逃逸作用。

### 1.1 肿瘤抗原

肿瘤抗原是细胞癌变过程中所出现的新抗原（neoantigen）及过度表达的抗原物质的总称，到目前已经发现肿瘤抗原近3, 000种。根据特异性可分为肿瘤特异抗原（tumor specific antigen, TSA）和肿瘤相关抗原（tumor-associated antigen, TAA）；根据肿瘤抗原产生机制分为突变基因或癌基因表达产物、异常表达的细胞蛋白、化学或物理因素诱发的肿瘤抗原、病毒诱发的肿瘤抗原、自发性肿瘤抗原、胚胎抗原和组织特异性分化抗原。

### 1.2 机体抗肿瘤免疫机制

对免疫原性强的肿瘤，特异性免疫应答是主要的；而对免疫原性弱的肿瘤，非特异性免疫应答可能具有更重要的意义。细胞免疫在免疫监视和抗肿瘤效应中占主导地位，体液免疫仅在某些情况下起协同作用，包括激活补体系统溶解肿瘤细胞、抗体依赖的细胞介导的细胞毒作用（antibody-dependent cell-mediated cytotoxicity, ADCC）、抗体的调理作用和抗体使肿瘤细胞的黏附特性改变或丧失。

#### 1.2.1 细胞免疫的效应细胞

细胞免疫的效应细胞有T细胞、自然杀伤（natural killer, NK）细胞、巨噬细胞（macrophage, Mφ）、髓系来源抑制细胞（myeloid-drived suppressor cells, MDSCs）。

T细胞分为主要组织相容性复合体（major histocompatibility complex, MHC）Ⅱ类抗原限制的辅助性T淋巴细胞（helper T cell, Th）或CD4^+^ T细胞和MHC Ⅰ类抗原限制的细胞毒T淋巴细胞（cytotoxic T lymphocyte, Tc）或CD8^+^ T细胞两种亚群。根据其分泌细胞因子的不同，CD4^+^ T细胞、CD8^+^ T细胞又分别分为Th1、Th2和Tc1、Tc2细胞亚群。Th1、Tc1细胞参与抗肿瘤细胞免疫效应，主要分泌白介素2（interleukin-2, IL-2）、γ干扰素（interferon-γ, IFN-γ）、肿瘤坏死因子α（tumor necrosis factor α, TNF-α）等激活单核-巨噬细胞、NK细胞，并增强CD8^+^ T细胞杀伤功能。Th2、Tc2细胞主要分泌IL-4、IL-5、IL-10等Ⅱ类因子，主要参与体液免疫。IFN-γ和IL-4能分别对Th1和Th2产生促生成作用，因此可以通过监测IFN-γ/IL-4的比例间接反映Th1/Th2水平。CD8^+^ T细胞是抗肿瘤免疫的主要效应细胞，其杀伤肿瘤细胞的机制有：①通过其抗原受体识别肿瘤细胞上的肿瘤抗原并与之结合，通过溶细胞作用，直接杀伤肿瘤细胞；②通过分泌多种细胞因子（如IFN-γ、TNF）间接杀伤肿瘤细胞。调节性T细胞（regulatory T cells, Treg）属于抑制性T细胞（suppressor T cells, Ts），它是一个异质性群体，主要来自CD4^+^CD25^+^表型细胞，在胸腺产生，旨在保持内环境稳定和免疫沉默，是一个重要的自身核对，主要包括：①通过分泌免疫抑制因子抑制效应细胞的功能，分泌免疫抑制因子如IL-10和转化生长因子β（transforming growth factor β, TGF-β）完成，其中TGF-β可以阻断T细胞和B细胞的活性、NK和淋巴因子激活杀伤细胞（lymphokine activated killer cells, LAK）和抗原呈递；②通过颗粒酶和穿孔素直接杀伤效应细胞；③通过干扰细胞代谢影响效应细胞的功能；④通过影响树突状细胞（dendritic cell, DC）的功能进而影响T细胞的活化以及Treg的诱生和增殖。Tregs表达CD4^+^CD25^+^，因此可以通过监测CD4^+^CD25^+^双阳性细胞水平来间接反映Tregs在消融后患者血清中的变化。

NK细胞占循环淋巴细胞的15%，能产生IFN-7、TNF-8等丰富的细胞因子，是细胞免疫中的非特异性成分。它不依赖抗体或补体、不需要预先活化即可直接杀伤肿瘤细胞，其杀伤作用不受肿瘤特异性和MHC限制，处于抗肿瘤的第一道防线。

巨噬细胞不仅是溶解肿瘤细胞的效应细胞，也是机体抗肿瘤细胞免疫中的抗原呈递细胞（antigen-presenting cells, APC）。

MDSCs来源于骨髓祖细胞和未成熟髓细胞，是树突状细胞、巨噬细胞和/或粒细胞的前体，具有CD34^+^CD33^+^CD13^+^CD15^-^ Treg的表型细胞。具有抑制T细胞功能和调节天然抗肿瘤免疫的作用。

#### 1.2.2 APC

APC包括DC、巨噬细胞、B细胞等，其中DC是功能最强的APC。DC表达MHC Ⅰ类、MHC Ⅱ类分子及T细胞活化所必需的共刺激分子，通过MHC Ⅰ类、MHC Ⅱ类分子及交叉呈递功能，捕获抗原，经其加工、处理后将抗原信息呈递给T、B细胞，分别刺激CD8^+^ T细胞和CD4^+^ T细胞。MHC Ⅰ类分子主要来源于自身抗原或病毒感染后细胞抗原，MHC Ⅱ类分子主要来源于外界抗原。巨噬细胞和DC主要区别在于交叉呈递能力不同。DC可将MHC Ⅱ类抗原交叉呈递进入MHC Ⅰ类途径，进而产生CD8^+^ T细胞。

### 1.3 肿瘤免疫逃逸

肿瘤免疫逃逸机制包括免疫抑制和免疫耐受。

免疫抑制是肿瘤诱导产生免疫抑制细胞，如Treg、MDSCs、肿瘤相关巨噬细胞、不成熟DC等。免疫抑制因子包括TGF-β、IL-10、前列腺素（prostaglandin E2, PGE2）、环氧合酶2、吲哚胺-2, 3-双加氧酶（indoleamine 2, 3-dioxygenase, IDO）、诱导型一氧化氮合酶（inducible nitric oxide synthase, iNOS）及Toll样受体（Toll-like receptor, TLR）等。

免疫耐受包括肿瘤局部的“免疫黑洞”和宿主免疫系统异常，前者表现为肿瘤细胞的抗原缺失和抗原调变，肿瘤抗原的封闭、遮蔽与隔离，肿瘤细胞MHC Ⅰ类、MHC Ⅱ类分子表达低下，肿瘤细胞共刺激信号缺乏，肿瘤细胞漏逸，免疫检查点和肿瘤细胞抗凋亡；后者表现为宿主血清中存在一定量的“增强抗体”，机体免疫抑制性细胞异常活跃，宿主APC功能低下或数量异常，肿瘤局部浸润淋巴细胞功能异常，外周血红细胞免疫状态改变，血清可溶性IL-2受体水平异常增高，外周血NK细胞活性降低和外周血Th1/Th2、Tc1/Tc2细胞比例失衡。

## 2 肺部肿瘤热消融

### 2.1 热消融技术

肺部肿瘤热消融技术主要有射频消融、微波消融和冷冻消融三种^[8]^。靶肿瘤消融后，可以诱发远处的肿瘤消退，这一现象称之为异位效应（abscopic effect）。

### 2.2 共同的免疫学机制^[9-13]^

消融中心区损伤相关分子模式（damage associated molecular patterns, DAMPs）是组织或细胞受到损伤、缺氧、应激等因素刺激后释放到细胞间隙或血液循环中的一类物质，主要包括：DNA、RNA、高迁移率族蛋白1（high mobility group protein 1, HMGB1）、热休克蛋白（heat shock proteins, HSPs）、钙网蛋白（calreticulin, CRT）、腺嘌呤核苷三磷酸（adenosine triphosphate, ATP）等物质作为危险信号与模式识别受体（pattern recognition receptor, PRR）被机体的免疫系统识别结合，激活多种免疫细胞诱导免疫反应。该区域积聚的DAMPs充当肿瘤抗原的重要储存库，逐渐流向区域淋巴结，促进DC细胞成熟，活化T细胞，激活机体抗肿瘤免疫。当细胞死亡发生时，“第一反应者”通常是非特异性免疫系统的代表，包括中性粒细胞、巨噬细胞和NK细胞。激活特异性免疫反应有四个要求：抗原呈递、T细胞的抗原识别、共刺激分子的相互作用和危险信号的持续存在。共刺激是指T细胞上的CD28和DC上的B7分子的非抗原特异性标志。热消融术后1.5 h-8 wk机体血清中的促炎性细胞因子出现暂时性的升高，引起明显的体温升高伴肾上腺素水平升高，但不导致严重的全身性炎症反应综合征（systemic inflammatory response syndrome, SIRS）与多器官功能衰竭和凝血功能障碍，但是体温、平均动脉压、血清中肾上腺素、去甲肾上腺素、C反应蛋白增加。

致死性高热的常用阈值是50 ℃。高于50 ℃的温度，由蛋白质变性引起的凝固性坏死基本上立即发生；低于50 ℃的温度，细胞死亡需要持续暴露。如果没有实现立即细胞死亡，则在恢复到常温条件后可以观察到由于热诱导的细胞损伤引起的延迟响应。在这种情况下，细胞死亡可以通过细胞凋亡发生，也可能由血管血栓形成导致组织缺血或再灌注损伤。消融过渡区由热介导的溶酶体激活或线粒体损伤而不是坏死，细胞死亡可能是细胞凋亡，或者可以发生细胞恢复。凋亡细胞不释放细胞内容物，被DC细胞摄取后无“危险信号”释放，不能有效刺激DC细胞成熟，致使T细胞活化受限，无法形成有效的免疫应答，甚至引起抑制性免疫反应。

肿瘤细胞坏死与凋亡的平衡是热消融治疗后免疫应答中的一个关键但很难表征（poorly characterized）的因素。正调节因素在细胞坏死时出现，包括炎性因子（IL-1、IL-6、IL-8、IL-12、TNF-α、IFN-γ）、免疫细胞因子（CD40L/CD40）、死亡肿瘤释放内源性佐剂（CDN、ATP、HMGB1）、肠道微生物产物增加。负调节在细胞凋亡时出现，包括IL-10、IL-4、IL-13升高。

### 2.3 各自的免疫学机制

射频消融^[14-17]^产热区域只在电极附近几毫米，热量通过热传导方式缓慢向外传递，对微小血管损伤小，免疫细胞能更好地通过血管进入肿瘤。射频消融可以引起大量促炎性细胞因子升高，消融后数小时出现，持续数天。引起Treg（CD4^+^CD25^+^Foxp3^+^）细胞数量减少，抑制性免疫反应减少，促进抗肿瘤免疫。射频消融也被证明会引起缺氧驱动的远离治疗部位（细胞凋亡）的转移性肿瘤生长。

微波消融引起的免疫效应与射频消融类似，但是由于肿瘤抗原变性及凝固性坏死更彻底，不利于抗肿瘤免疫效应激活，因此免疫效应较射频消融弱。

冷冻消融^[18, 19]^可以引起的液化性坏死较凝固性坏死能更好地保存肿瘤抗原免疫原性，并且有利于DAMPs释放以及免疫细胞浸润、抗原呈递及T细胞活化。冷冻比热消融更能引起大量促炎性细胞因子升高，消融后数小时出现，持续数天。免疫效应是激活还是抑制取决于肿瘤细胞坏死-凋亡的比例及Treg产生多少有关。

极高温度诱发不可逆转的细胞损伤和凝固组织坏死。肿瘤抗原多变性，免疫原性差，凝固性坏死不利于DAMPs的释放及免疫细胞的浸润，因此热消融免疫效应较冷冻消融弱^[10, 13]^。

## 3 肿瘤热消融联合肿瘤免疫

### 3.1 肺部肿瘤热消融存在的问题

消融后循环肿瘤细胞增加是否促进肿瘤转移^[20]^？消融过渡区肿瘤细胞发生凋亡（缺乏有效抗原释放，对宿主免疫系统通常没有损害）是否促进肿瘤进展复发^[21-23]^？

热消融产生的抗肿瘤免疫异位效应通常较弱，无法完全清除肿瘤细胞^[19, 24]^。为此需要联合免疫治疗，降低转移和复发。

### 3.2 肿瘤免疫治疗

1868年，Wilhelm Busch首次报道使用丹毒感染癌症患者后肿瘤显著缩小。1891年，美国纽约纪念医院骨科医师William Coley开始用细菌来治疗肿瘤的试验，经历了漫长的探索，遭遇重大挫折。直到1984年，美国国立癌症研究院Steve Rosenberg团队成功地用高剂量IL-2治愈1例患者，给肿瘤免疫治疗带来一线曙光。此后，包括单克隆抗体、肿瘤疫苗不断涌现，虽然对一些患者有效，但未被大规模应用。

特异性主动免疫治疗主要是指肿瘤疫苗，通过给患者体内导入肿瘤抗原，激发患者的特异性抗肿瘤免疫反应，具有特异性强、持续时间长等优点。比利时学者Thierry Boon教授长期研究癌细胞抗原问题。近年来，已经涌现出细胞疫苗、全蛋白疫苗、多肽疫苗、核酸疫苗、重组病毒疫苗、基因修饰的瘤苗（将某些细胞因子基因、辅助刺激分子基因、MHC Ⅰ类抗原分子基因等转移入肿瘤细胞后，可降低其致瘤性，增强其免疫原性）、抗独特性抗体瘤苗（抗独特性抗体是抗原的内影像，可以模拟肿瘤抗原成为瘤苗）、DC疫苗等。

特异性被动免疫治疗包括单克隆抗体的免疫治疗[人类表皮生长因子受体2（human epidermal growth factor receptor 2, HER2）、血管内皮生长因子（vascular endothelial growth factor, VEGF）、细胞毒性T淋巴细胞相关蛋白4（cytotoxic T lymphocyte associated protein 4, CTLA-4）、程序性死亡受体1（programmed cell death protein 1, PD-1）/程序性死亡配体1（programmed cell death ligand 1, PD-L1）]、抗体导向药物治疗[过继细胞免疫：巨噬细胞、NK细胞、LAK细胞、细胞因子诱导的杀伤细胞（cytokine-induced killer, CIK）细胞、肿瘤浸润淋巴（tumor infiltrating lymphocyte, TIL）细胞、细胞毒性T淋巴（cytotoxic T lymphocyte, CTL）细胞、嵌合抗原受体T细胞免疫疗法（chimeric antigen receptor T, CAR-T）细胞和T细胞受体基因工程改造的T细胞（T cell receptor-gene engineered T, TCR-T）细胞]、细胞因子[ILs类：IL-2、IL-12；IFNs类IFN-α、IFN-β、IFN-γ；集落刺激因子（colony stimulating factor, CSF）类：粒细胞集落刺激因子（granulocyte CSF, G-CSF）、粒细胞-巨噬细胞集落刺激因子（granulocyte-macrophage CSF, GM-CSF）；TNFs类：TNF-α、TNF-β；TGFs类：TGF-α、TGF-β）]。

非特异性免疫治疗包括微生物及有关成分（卡介苗BCG、短小棒状杆菌CP、溶血性链球菌制剂OK-432等）、多糖类（酵母多糖、云芝多糖PSK、香菇多糖Lentinan等）、胸腺肽α1、合成免疫调节剂（如聚肌胞苷酸、CpG-寡脱氧核苷酸CpG-ODN、左旋咪唑等）和重组人体乳转铁蛋白（talactoferrin, TLF）。

免疫检查点抑制剂（immune checkpoint inhibitor, ICI）的疗效主要与以下因素相关：肿瘤突变负荷（tumor mutation burden, TMB）（≥20、6-19、≤5）、微卫星高度不稳定（microsatellite instability-high, MSI-H）、错配基因修复缺失（mismatch-repair deficiency, MMR）、肿瘤微环境中的PD-L1表达丰度及肿瘤局部浸润淋巴细胞数量、肠道微生态、*KRAS*突变（同时携带*KRAS*和*P53*突变的患者对PD-1治疗敏感；同时携带*KRAS*和*STK11*突变的患者对PD-1治疗抵抗；其他患者对PD-1治疗的有效率居中）。

### 3.3 联合治疗

热消融作为微创且精准的肿瘤治疗技术，能否通过联合肿瘤免疫治疗提高协同作用是目前研究的热点。多项研究证实热消融联合DC或免疫佐剂（CpG-ODN、OK-432、白介素、GM-CSF、趋化因子和TLR激动剂）^[25-32]^、过继免疫细胞（DC-CIK及NK细胞）^[33, 34]^、CTLA-4单抗^[35]^、PD-1/PD-L1抗体^[36]^、Treg耗竭^[35]^等可以促使机体产生更加强大的抗肿瘤免疫效应，从而获得更好的疗效。比如患者生活质量的改善，CD3、CD4、CD8、CD4/CD8等免疫指标的提升等，说明热消融联合免疫治疗具有协同作用。但是上述研究仍处于初步研究阶段，临床效果有待于进一步研究。

总之，热消融联合免疫治疗能否提高肺部肿瘤患者的生存率？热消融联合免疫治疗能否提高完全消融率？哪种消融联合免疫治疗最有效？等等问题还需要进一步探讨。目前正在进行的Ⅰ期/Ⅱ期临床注册研究或许能够回答这些疑问，免疫治疗主要是针对PD-1/PD-L1，热消融技术主要有冷冻、微波等，这些研究有NCT02469701、NCT02843815、NCT03290677、NCT03769129、NCT04049474、NCT04102982、NCT04201990、NCT04339218等，希望能尽快取得喜人的结果并指导临床治疗。
